# The receptor BLT1 is essential on neutrophils in a mouse model of mucous membrane pemphigoid

**DOI:** 10.1172/jci.insight.173914

**Published:** 2025-09-23

**Authors:** Tabea Bremer, Sripriya Murthy, Sabrina Patzelt, Paul Schilf, Mareike Neumann, Sina Gonther, Jasper Pruessmann, Wiebke Pruessmann, Enno Schmidt, Thomas Rülicke, Christian D. Sadik

**Affiliations:** 1 Department of Dermatology, Allergy, and Venereology and; 2 Lübeck Institute of Experimental Dermatology (LIED), University of Lübeck, Lübeck, Germany.; 3 Department of Biomedical Sciences and Ludwig Boltzmann Institute for Hematology and Oncology, University of Veterinary Medicine Vienna, Vienna, Austria.

**Keywords:** Autoimmunity, Dermatology, Autoimmune diseases, G protein-coupled receptors, Neutrophils

## Abstract

Mucous membrane pemphigoid (MMP) is a mucocutaneous autoimmune blistering disease affecting diverse mucous membranes and the skin with inflammatory blisters and erosions. The pathogenesis of MMP is only poorly understood, but inflammation in MMP is triggered by specific binding of autoantibodies directed to different proteins of the dermal-epidermal/-epithelial junction, subsequently leading to the influx of inflammatory cells, particularly neutrophils, into the dermis. Using the anti-laminin 332 antibody transfer model of MMP, we addressed the molecular mechanisms of neutrophil infiltration and its significance for the eruption of mucocutaneous lesions. Mice deficient in 5-lipoxygenase (*Alox5^–/–^*) or in the leukotriene B_4_ (LTB_4_) receptor BLT1 (*Ltb4r1^–/–^*) were resistant to skin inflammation and exhibited substantially fewer mucosal lesions, with deficiency in either gene compromising the recruitment of neutrophils to the lesion. Furthermore, neutrophil-specific genetic deficiency in *Ltb4r1* similarly protected from MMP. Hence, BLT1 was required on neutrophils, and neutrophil recruitment was indispensable for the eruption of lesions in MMP. In line with these findings, the BLT1 inhibitor CP-105,606 ameliorated MMP dose-dependently. Collectively, our results highlight neutrophils and LTB_4_/BLT1 as key drivers of inflammation in MMP and as promising therapeutic targets.

## Abstract 2

The lipid mediator leukotriene B_4_ is drives mucocutaneous inflammation in mucous membrane pemphigoid by mediating the recruitment of neutrophils into the skin and mucosal surfaces.

## Introduction

Pemphigoid diseases are a group of autoimmune blistering skin diseases. They affect the skin and diverse mucosal surfaces with subepidermal blisters and erosions because of an autoantibody-driven immune response against different proteins of the dermal-epidermal adhesion complex (1, 2). Mucous membrane pemphigoid (MMP) affects primarily mucosal surfaces, including those of the conjunctiva, the oral cavity, the esophagus, the nose, the pharynx, the larynx, the trachea, the anal canal, and the genitalia. It can lead to scarring and, consequently, to debilitating residual states, including blindness and strictures of the respiratory and upper gastrointestinal tract (1, 3–5).

MMP is a clinically and immunopathologically heterogenous disease. Autoantigens described in MMP include the C-terminus of type XVII collagen (BP180), BP230, laminin 332, integrin α6β4, and type VII collagen, with BP180 and laminin 332 as the major autoantigens (6–8). Laminin 332 is an extracellular glycoprotein binding to the integrins α6β4 and α3β1 in the basement membrane zone of epithelia (9, 10). It is composed of 3 subunits, of which the α3 subunit is the most frequent autoantigen in laminin 332 MMP (11, 12). Autoantibodies against the α3 chain of laminin 332 are prevalent in 40%–50% of patients with MMP (9, 13) and have been reported to be associated with a more aggressive course of disease (11, 14).

Histopathologically, both mucosal and cutaneous lesions exhibit a mixed inflammatory infiltrate, with neutrophils usually constituting the largest cell population (15). Neutrophils are believed to be critical for the emergence of tissue inflammation and to degrade the dermal-epidermal adhesion complex, thus driving the formation of subepidermal clefts.

Recently, a novel mouse model mimicking the effector phase of MMP has been developed (16). The model is based on the application of rabbit IgG directed to the α3 chain of laminin 332. This elicits cutaneous and mucosal lesions at ocular, oral, laryngeal, and pharyngeal surfaces clinically and histopathologically closely resembling those in patients with MMP (16–18). Neutrophils dominate the dermal infiltrate in this model and have been surmised to play a pivotal role for the eruption of inflammatory lesions. However, this hypothesis has not been tested, and the chemoattractant(s) guiding neutrophils into the dermis are elusive.

In general, neutrophils can express more than 30 chemoattractant receptors, including the anaphylatoxin receptors C5aR1 and C5aR2, the chemokine receptors CXCR2 and CCR1, the formyl peptide receptors FPR1 and FPR2, as well as the leukotriene B_4_ (LTB_4_) receptor BLT1 (19–22). Thus, they are responsive to a plethora of chemoattractants (23). We have recently demonstrated that the immunomodulatory drug dapsone, which is a first-line treatment of moderate MMP, also ameliorates disease in our MMP mouse model (18). Dapsone bears multiple pharmacological activities, including inhibition of 5-lipoxygenase, the key enzyme in the biosynthesis of all leukotrienes and of BLT1 (18). Our results therefore hinted at a role of LTB_4_/BLT1 in the regulation of tissue inflammation in MMP, but the significance of LTB_4_/BLT1 for the pathogenesis of MMP has not been addressed. Here, we therefore examined the contribution of LTB_4_/BLT1 to disease in the anti-laminin 332 (anti-LAMA332) IgG antibody transfer mouse model of MMP.

## Results

### Genetic deficiency in 5-lipoxygenase or BLT1 protects from both cutaneous and mucosal lesions in MMP.

**Figure 1:**
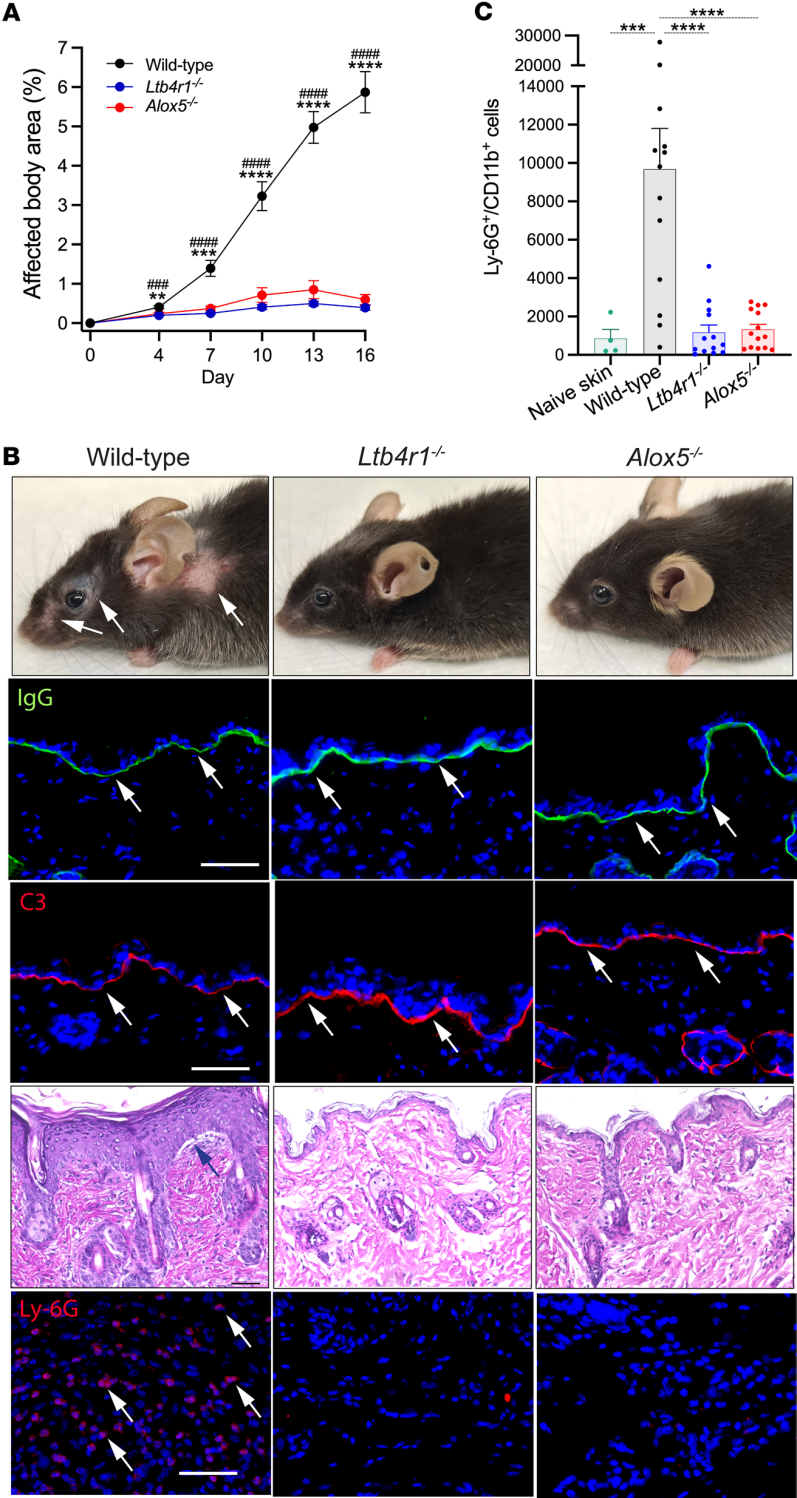
Genetic deficiency in 5-lipoxygenase or BLT1 confers resistance to the eruption of inflammatory skin lesions in MMP. (**A**) Percentage of the total body surface area affected by inflammatory skin lesions in wild-type, *Ltb4r1^–/–^*, and *Alox5^–/–^* mice from day 0 to day 16. (**B**) Representative pictures of wild-type, *Ltb4r1^–/–^*, and *Alox5^–/–^* mice on day 16. The individual panels show (a) clinical presentation, arrows indicate inflammatory skin lesions; (b) direct immunofluorescence staining for IgG, arrows indicate the linear deposition of IgG at the dermal-epidermal junction; (c) immunofluorescence staining for C3, arrows indicate the deposition of C3 at the dermal-epidermal junction; (d) H&E stainings for inflammatory skin lesions in wild-type mice and of corresponding skin sites in *Ltb4r1^–/–^* and *Alox5^–/–^* mice, arrow indicates dermal-epidermal clefts; (e) immunofluorescence stainings for Ly-6G in the dermis of inflamed skin, arrows indicate examples for Ly-6G^+^ cells. (**C**) Number of neutrophils (Ly-6G^+^CD11b^+^ cells) recovered from a 6 mm punch biopsy of inflamed skin in wild-type mice or of corresponding body sites of healthy wild-type mice (Naive skin) and *Ltb4r1^–/–^* and *Alox5^–/–^* mice assessed by flow cytometry. All results are presented as mean ± SEM. Each dot in **C** represents a mouse. Results in **A** were compared by 2-way ANOVA and Holm-Šídák post hoc test (*n* = 9 male and 9 female mice/group). **P < 0.01; ***P < 0.001; ****P < 0.0001 for the comparison between wild-type and *Alox5^–/–^* mice; ^###^*P* < 0.001; ^####^*P* < 0.0001 for the comparison between wild-type and *Ltb4r1^–/–^* mice. Results in **C** were compared by 1-way ANOVA and Holm-Šídák post hoc test (*n* = 4–14 mice/group). ***P < 0.001; ****P < 0.0001 for the comparisons indicated.

To elucidate the significance of leukotrienes in general and specifically of LTB_4_ for the effector phase of anti-LAMA332 MMP, wild-type mice and mice deficient in the genes encoding either 5-lipoxygenase (*Alox5*) or BLT (*Ltb4r1*) were treated with anti-LAMA332 IgG, as described in detail in the Methods section. In wild-type mice, this treatment induced the eruption of skin lesions by day 7 (Figure 1A). The percentage of the total body surface affected by skin lesions continuously increased throughout the entire observation period of 16 days. On day 16, approximately 6% of the total body surface of wild-type mice was affected (Figure 1, A and B). In sharp contrast, both *Alox5^–/–^* and *Ltb4r1^–/–^* mice did not develop any skin lesions throughout the experiment (Figure 1, A and B). This complete protection was not due to a comprised deposition of anti-LAMA332 IgG and C3 at the dermal-epidermal junction in *Ltb4r1^–/–^* and *Alox5^–/–^* mice (Figure 1B). H&E and immunofluorescence stainings for the neutrophil cell surface marker Ly-6G revealed that neutrophils were only recruited into the dermis in wild-type mice but not in *Ltb4r1^–/–^* and *Alox5^–/–^* mice (Figure 1B).

We also quantified the number of neutrophils in 6 mm punch biopsies from the skin of wild-type mice left untreated, lesional skin from wild-type mice, and corresponding skin sites from *Alox5^–/–^* and *Ltb4r1^–/–^* mice subjected to anti-LAMA332 IgG and harvested on day 16 by flow cytometry. The gating strategy is shown in Supplemental Figure 1; supplemental material available online with this article; https://doi.org/10.1172/jci.insight.173914DS1 This analysis verified that neutrophils abundantly infiltrate lesional skin of wild-type mice whereas they are hardly found in naive skin of wild-type mice (Figure 1C). The number of neutrophils in the skin of *Ltb4r1^–/–^* and *Alox5^–/–^* mice equaled the number in the skin of untreated wild-type mice, indicating that there is no recruitment of neutrophils at all into the skin in *Ltb4r1^–/–^* and *Alox5^–/–^* mice.

**Figure 2:**
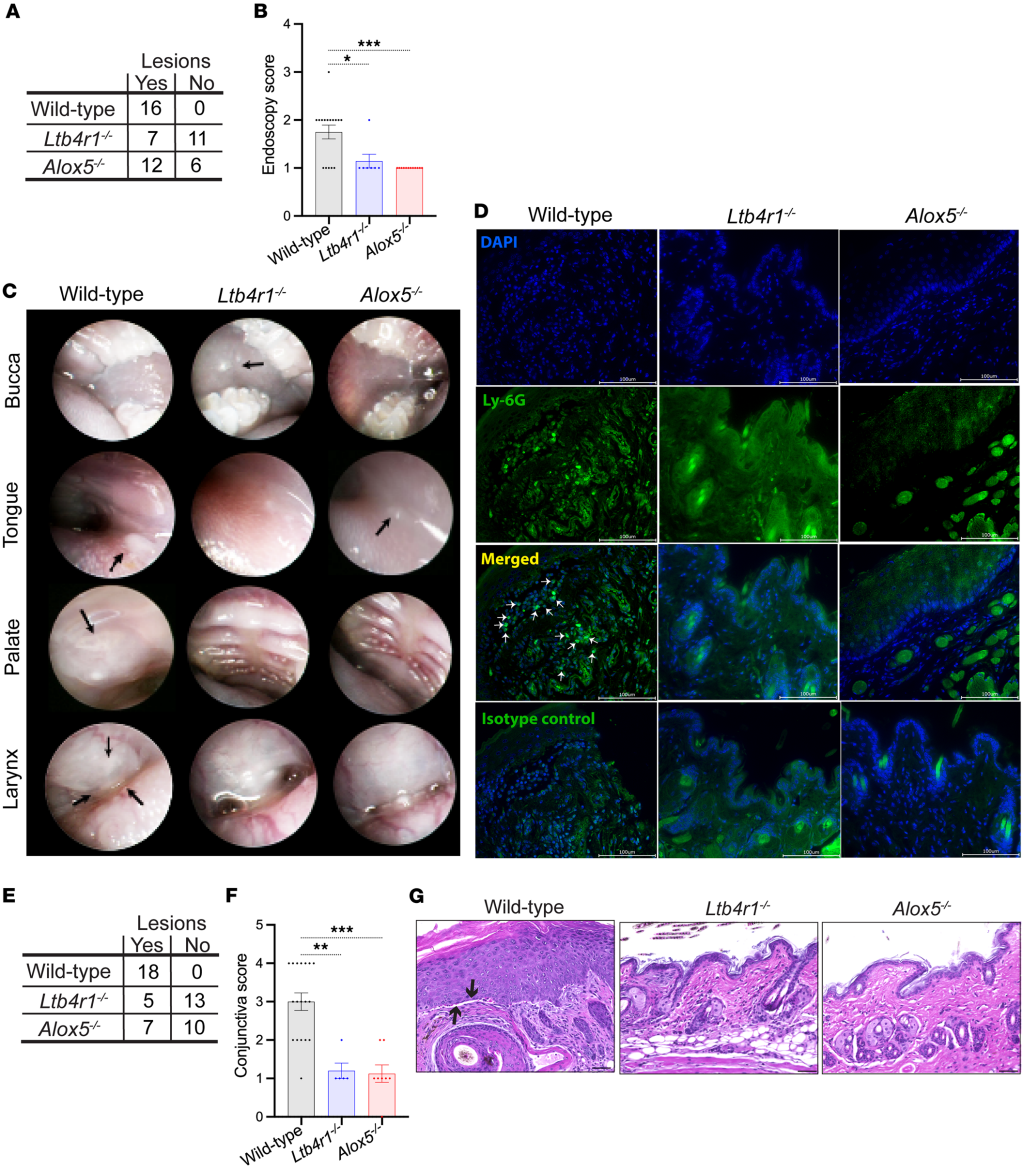
Oropharyngeal and conjunctival mucosal lesions are reduced in *Alox5^–/–^* and *Ltb4r1^–/–^* mice. The oropharyngeal space was examined for mucosal lesions by endoscopy on day 16. (**A**) Contingency table of the number of wild-type, *Ltb4r1^–/–^*, and *Alox5^–/–^* mice with and without lesions (8–9 male and 8–9 female mice/group). (**B**) Comparison of the severity of mucosal lesions in the oropharynx evaluated with the endoscopy score. In this analysis, only those mice exhibiting at least 1 lesion were included (*n* = 7–16 mice/group). (**C**) Representative endoscopy pictures of the mucosa of the cheek, tongue, palate, and larynx in wild-type, *Ltb4r1^–/–^*, and *Alox5^–/–^* mice. Arrows indicate inflammatory lesions. (**D**) Immunofluorescence staining for Ly-6G of lesions of the buccal mucosa taken on day 16. Arrows indicate Ly-6G^+^ cells on the Ly-6G/DAPI merged images. Scale bars equal 100 μm. Conjunctivitis was assessed by histopathology of specimens taken on day 16. (**E**) Contingency table of the number of wild-type, *Ltb4r1^–/–^*, and *Alox5^–/–^* mice with and without conjunctival lesions (8–9 male and 9 female mice/group). (**F**) Severity of conjunctivitis assessed by the conjunctival score. The comparison only includes those mice with conjunctivitis (*n* = 5–18). (**G**) Representative H&E staining of the conjunctiva on day 16. Arrows indicate dermal-epidermal clefts; scale bars equal 50 μm. The results in **B** and **F** are presented as mean ± SEM with each dot representing a mouse. The results in **A** and **E** were analyzed by χ^2^ test and in **B** and **F** by Kruskal-Wallis test with Dunn’s correction for multiple comparisons. The groups in **A** and **F** significantly differed in the frequency of mice developing lesions with *P* < 0.001. In **B** and **F**, *P < 0.05; **P < 0.01; ***P < 0.001 for the comparisons indicated.

To assess the involvement of mucosal surfaces, we determined the number of areas affected by inflammatory lesions in the oropharynx by endoscopy at the end of the experiment on day 16 (Figure 2A). Mucosal lesions had erupted in the oral cavity and the pharynx of all wild-type mice but only in 67% and 39% of *Alox5^–/–^* and *Ltb4r1^–/–^* mice, respectively (Figure 2A). The extent of lesions in those *Alox5^–/–^* and *Ltb4r1^–/–^* mice developing mucosal lesions was reduced in comparison with wild-type mice (Figure 2, B and C). More specifically, while wild-type mice reached a mean mucosal lesion score of 1.75, mucosal lesions in both *Alox5^–/–^* and *Ltb4r1^–/–^* mice only reached a score of 1.0; i.e., in most wild-type mice 2 quadrants and in *Alox5^–/–^* and *Ltb4r1^–/–^* mice only 1 quadrant of the oral cavity and pharynx bore lesions. Stainings for Ly-6G of skin biopsies taken from lesions in the buccal mucosa on day 16 confirmed the presence of neutrophils (Ly-6G^+^ cells) in the lamina propria of wild-type mice but not of *Alox5^–/–^* and *Ltb4r1^–/–^* mice (Figure 2D).

Furthermore, all wild-type mice but only 41% and 28% of *Alox5^–/–^* and *Ltb4r1^–/–^* mice, respectively, developed conjunctivitis (Figure 2, E and G), which, in those *Alox5^–/–^* and *Ltb4r1^–/–^* mice developing it, was also milder than in wild-type mice (Figure 2F).

### The BLT1 antagonist CP-105,696 ameliorates MMP.

**Figure 3:**
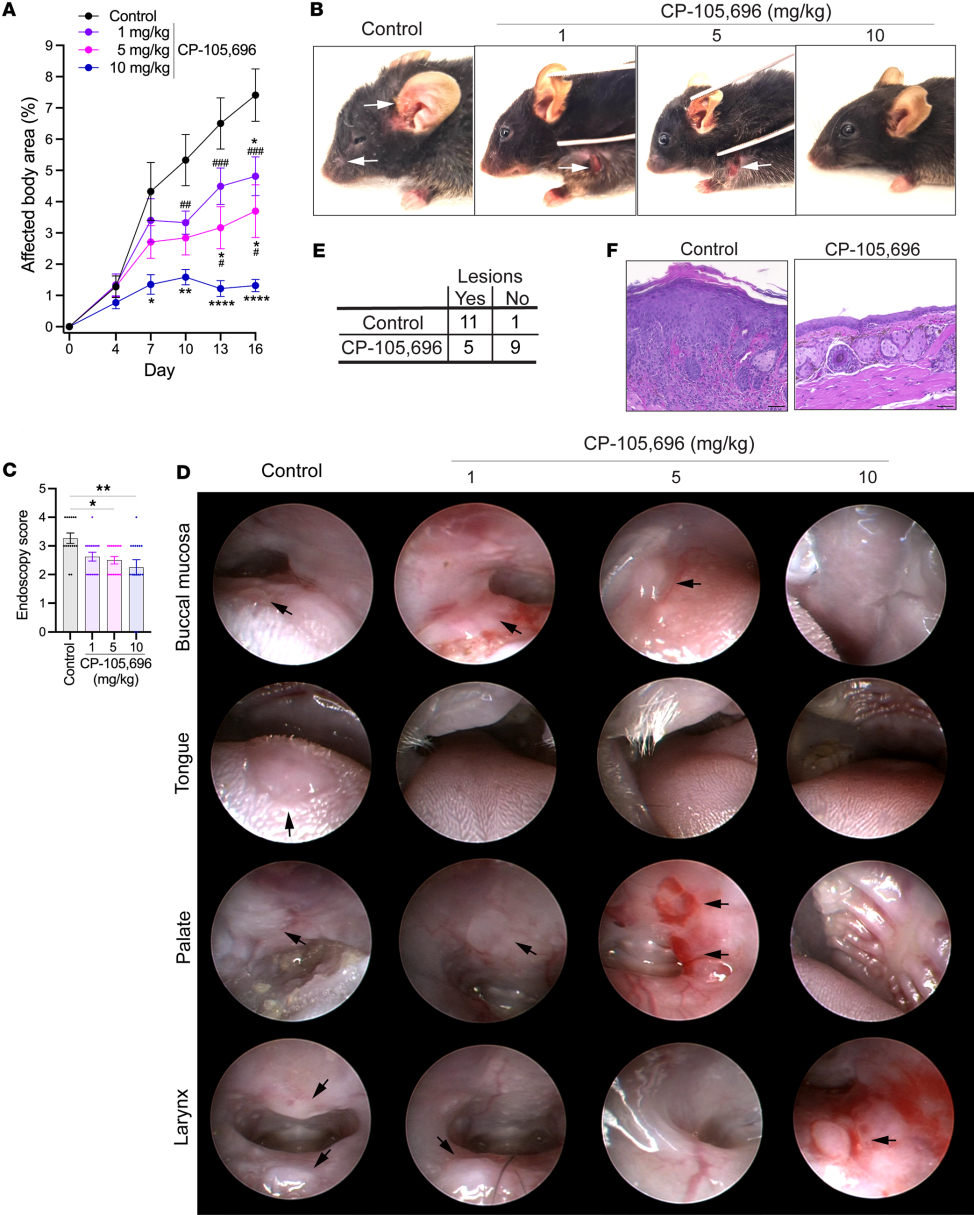
The BLT1 antagonist CP-105,696 ameliorates mucocutaneous inflammation in MMP dose-dependently. Wild-type mice were subjected to the MMP model and received 1, 5, or 10 mg/kg body weight CP-105,696 or its vehicle daily p.o. starting on day 0. (**A**) Progression of skin inflammation assessed by the percentage of the total body surface affected by skin lesions (*n* = 7–8 male and 8 female mice/group). (**B**) Representative pictures of all groups on day 13. Arrows indicate inflammatory lesions. (**C**) Comparison of the severity of mucosal lesions in the oropharynx evaluated by the endoscopy score (*n* = 7–8 male and 8 female mice/group). (**D**) Representative endoscopy pictures of mucosal surfaces on day 16. Arrows indicate inflammatory lesions. (**E**) Contingency table of the number of vehicle- and 10 mg/kg CP-105,696–treated mice with and without conjunctival lesions (7 male and 7 female mice/group). The frequency of mice with inflammatory lesions statistically significantly differed between the 2 groups with *P* < 0.01 determined by χ^2^ test. (**F**) Representative H&E staining of the conjunctiva of vehicle- and CP-105,696–treated mice on day 16. Scale bars equal 50 μm. All results are presented as mean ± SEM. In **C**, each dot represents a mouse. The results were examined for statistical significance in **A** by 2-way ANOVA and Holm-Šídák post hoc test and in **C** by Kruskal-Wallis test with Dunn’s correction for multiple comparisons. In **A**, at the days indicated, *P < 0.05; **P < 0.01; ****P < 0.0001 for the comparison of the indicated groups with the vehicle group; ^#^*P* < 0.05; ^##^*P* < 0.01; ^###^*P* < 0.001 for the comparison of the indicated group with the 10 mg/kg CP-105,696 treatment group. In **C**, *P < 0.05; **P < 0.01 for the comparisons indicated.

To further corroborate the potential of BLT1 as a pharmacological target in MMP, we assessed the effect of pharmacological inhibition of BLT1 in the MMP mouse model. For this purpose, MMP was induced in wild-type mice, which were treated with 1, 5, or 10 mg/kg body weight of the BLT1 inhibitor CP-105,696 or its vehicle daily by gavage. The administration of the drug was started with the first application of anti-LAMA332 IgG on day 0. CP-105,696 reduced the severity of skin inflammation in a dose-dependent manner (Figure 3, A and B). Even 1 mg/kg CP-105,696 exhibited a therapeutic effect, which further increased at the doses of 5 and 10 mg/kg CP-105,696 (Figure 3, A and B). The 10 mg/kg CP-105,696 dose ablated most of the skin inflammation. Similarly, in the oropharynx, CP-105,696 ameliorated mucosal inflammation dose-dependently starting at the dose of 1 mg/kg (Figure 3, C and D). The 10 mg/kg CP-105,696 dose decreased the number of quadrants affected by inflammatory lesions by 31%. We also examined the effect specifically of 10 mg/kg CP-105,696 on conjunctivitis, and this dose decreased the proportion of mice developing conjunctivitis (Figure 3, E and F).

**Figure 4:**
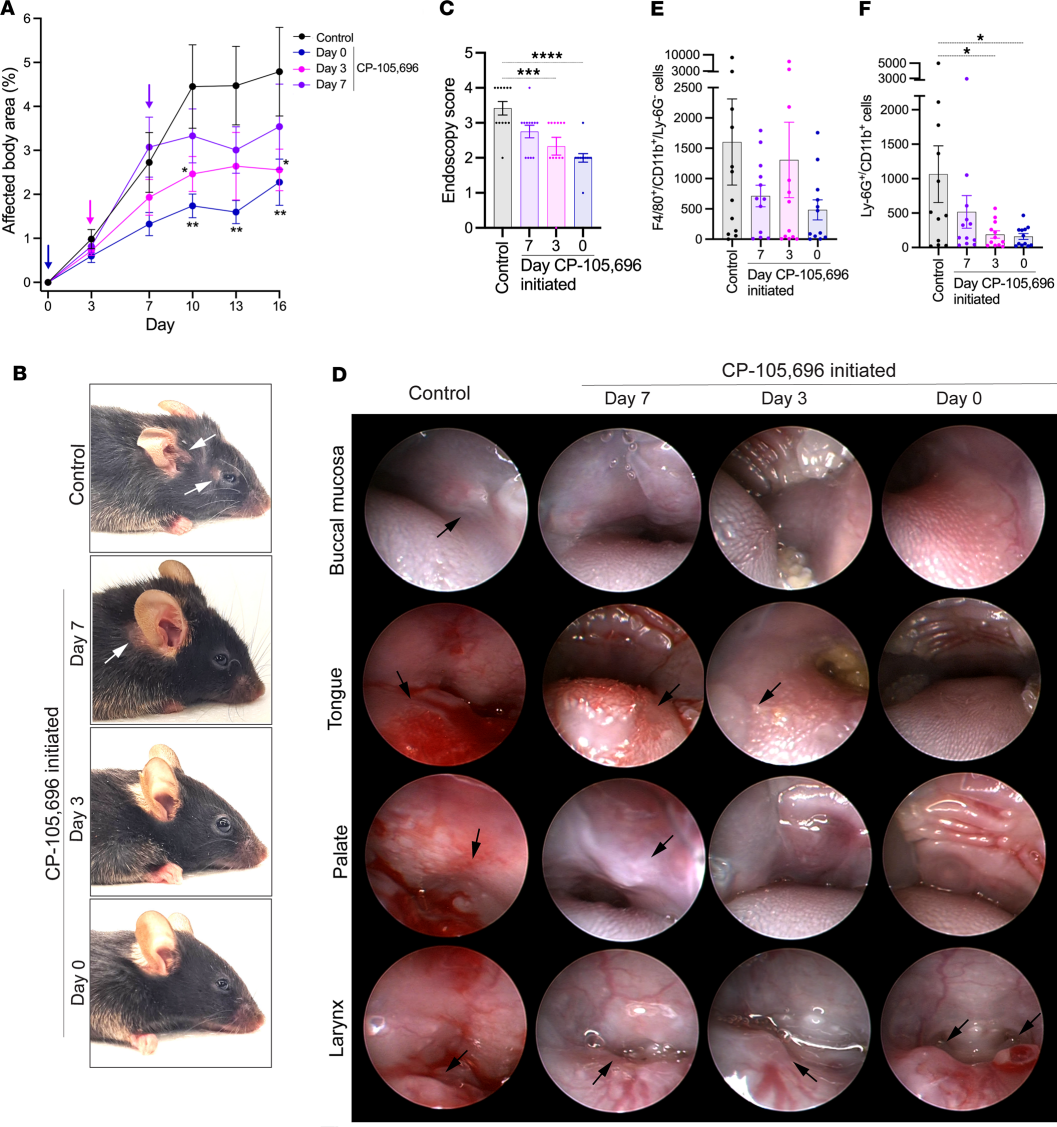
The BLT1 antagonist CP-105,696 disrupts the progression of mucocutaneous inflammation in the ongoing effector phase of MMP. Wild-type mice were subjected to the MMP model and were treated p.o. daily with 10 mg/kg body weight CP-105,696 or its vehicle starting on day 0, 3, or 7. (**A**) Progression of skin inflammation assessed by the percentage of the total body surface affected by skin lesions from day 0 through day 16 (*n* = 6 male and 6 female mice/group). Colored arrows indicate the days CP-105,696 treatment was initiated. (**B**) Representative pictures of vehicle and CP-105,696–treated mice on day 16. Arrows indicate inflammatory lesions. (**C**) Comparison of the extent of mucosal lesions in the oropharynx evaluated by the endoscopy score (*n* = 6 male and 6 female mice/group). (**D**) Representative endoscopy pictures of the presentation of the mucosa of the cheek, tongue, palate, and larynx in vehicle- and CP-105,696–treated mice. Arrows indicate inflammatory lesions. Number of (**E**) macrophages and (**F**) neutrophils, defined as F4/80^+^CD11b^+^Ly-6G^–^ and Ly-6G^+^CD11b^+^ cells, respectively, recovered from a 6 mm punch biopsy of lesional skin taken on day 16. Results are presented as mean ± SEM. All results are presented as mean ± SEM. In **C**, **E**, and **F**, each dot represents a mouse. The results were examined for statistical significance in **A** by 2-way ANOVA and Holm-Šídák post hoc test, in **C** by Kruskal-Wallis test with Dunn’s correction for multiple comparisons, and in **E** and **F** by 1-way ANOVA and Holm-Šídák post hoc test. In **A**, at the days indicated, *P < 0.05; **P < 0.01 for the comparison of the indicated groups to the control group. In **C**, **E**, and **F**, *P < 0.05; ***P < 0.001; ****P < 0.0001 for the comparisons indicated.

We next addressed whether 10 mg/kg CP-105,696 may still be effective in ameliorating disease if its administration is started after the establishment of the first inflammatory lesions. To this end, MMP was induced in wild-type mice, and daily treatment with 10 mg/kg CP-105,696 was started on day 0, 3, or 7. At all times initiated, treatment with CP-105,696 swiftly exerted an effect on skin inflammation, diluting its progression (Figure 4, A and B). When initiated on day 3 or 7, CP-105,696 still ameliorated oropharyngeal lesions by the end of the experiment on day 16 (Figure 4, C and D).

We also determined the number of neutrophils and macrophages in skin lesions on day 16 by flow cytometric analysis of single-cell suspensions. Treatment with CP-105,696 did not alter the number of macrophages (Figure 4E), but it decreased the number of neutrophils in the lesions independent of the time treatment was initiated (Figure 4F), hence, suggesting a continuous requirement of BLT1 signaling in the recruitment of neutrophils into the skin.

### LTB_4_/BLT1 drive the eruption of inflammatory lesions by direct action upon neutrophils.

**Figure 5:**
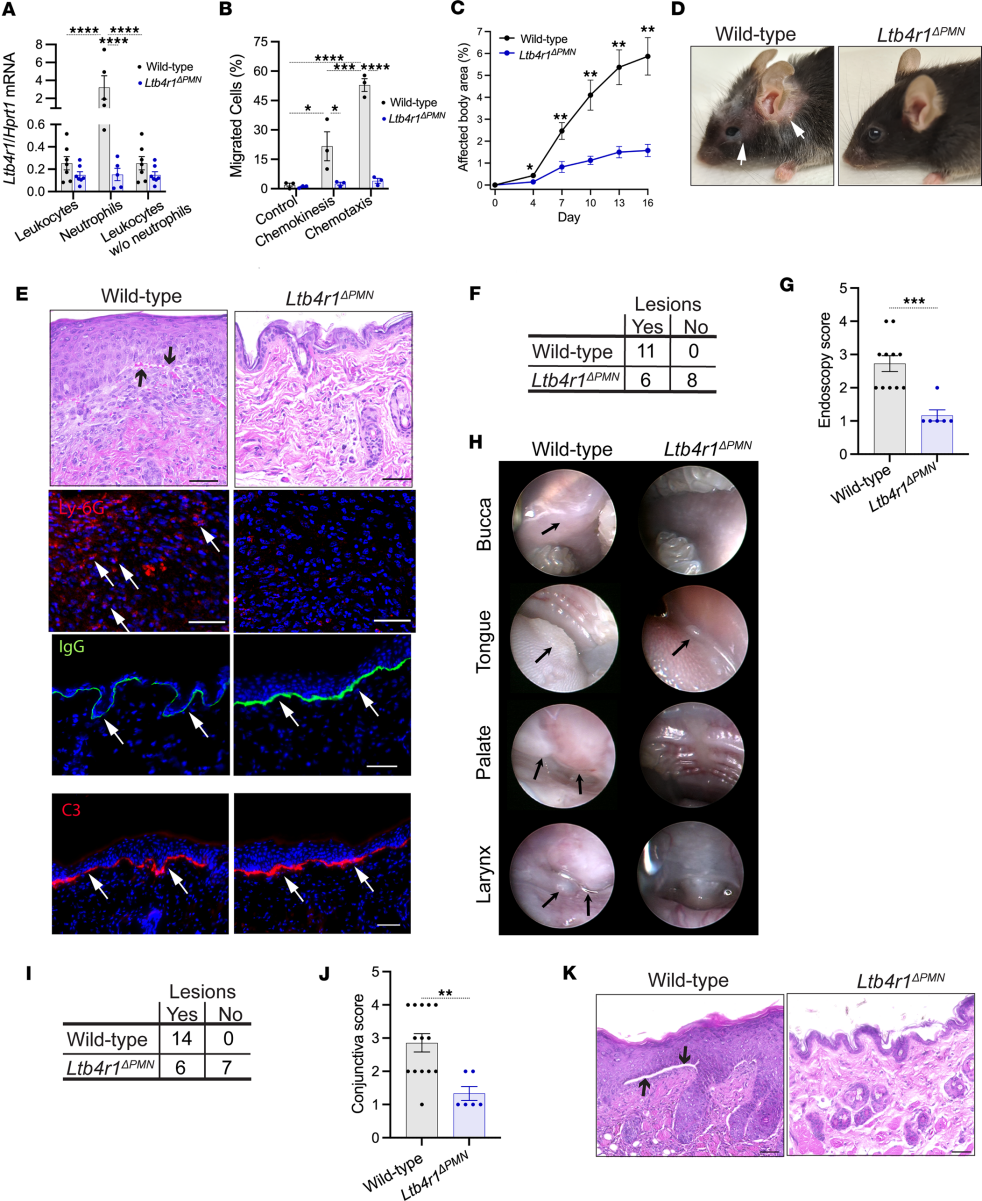
Neutrophil-specific *Ltb4r1^–/–^* (*Ltb4r1^ΔPMN^*) mice are protected from mucocutaneous lesions in the MMP model. (**A**) *Ltb4r1/Hprt1* mRNA levels in bone marrow leukocytes, neutrophils, and neutrophil-depleted leukocytes. (**B**) Chemotaxis of neutrophils toward LTB_4_. (**C**) The total body surface (%) affected by lesions (*n* = 7 male and 7 female mice/group). (**D**) Representative clinical presentation and (**E**) histopathology and immunopathology of the skin on day 16 with (a) H&E staining of lesional skin, arrows indicate dermal epidermal clefts; (b) immunofluorescence stainings for Ly-6G in the dermis of inflamed skin, arrows indicate Ly-6G^+^ cells; (c) direct immunofluorescence staining for IgG, arrows indicate the IgG depositions at the dermal-epidermal junction; and (d) immunofluorescence staining for C3, arrows indicate C3 depositions at the dermal-epidermal junction. (**F**) Contingency table of wild-type and *Ltb4r1^ΔPMN^* mice with and without oropharyngeal lesions. (**G**) Severity of mucosal lesions in those mice developing lesions (*n* = 6–11 mice/group). (**H**) Representative endoscopic pictures of mucosal surfaces. Arrows indicate inflammatory lesions. (**I**) Contingency table of wild-type and *Ltb4r1^ΔPMN^* mice with and without conjunctival lesions (*n* = 7 male and 6–7 female mice/group). (**J**) Severity of conjunctivitis in those mice with mucosal inflammation (*n* = 6–14). (**K**) Representative H&E stainings of the conjunctiva. Arrows indicate dermal-epidermal clefts, and scale bars equal 50 μm. Data merged from 2 independent experiments are presented as mean ± SEM. Each dot in **A**, **B**, **G**, and **J** represents a mouse. Results in **A**–**C** were compared by 2-way ANOVA and Holm-Šídák post hoc test and in **G** and **J** by Mann-Whitney test. *P < 0.05; **P < 0.01; ***P < 0.01; ****P < 0.0001 for the comparisons indicated. The results in **G** and **I** were analyzed by χ^2^ test. In both, the groups significantly differed with *P* < 0.01.

To understand the role of LTB_4_/BLT1 in MMP in more detail, we set out to identify its main target cell population in our model. BLT1 can be expressed on diverse immune and nonimmune cell populations (24), including neutrophils, which express the receptor constitutively on the surface of the entire cell population (19). With neutrophils dominating the cellular infiltrate in MMP, we hypothesized that BLT1 may be mainly required on neutrophils. We therefore generated neutrophil-specific *Ltb4r1^–/–^* (*Ltb4r1*^ΔPMN^) mice by the lox-cre technology using *MRP8-Cre-ires/GFP* mice. The effectivity of the deletion of *Ltb4r1* in neutrophils of *Ltb4r1*^ΔPMN^ mice was assessed at the mRNA level by reverse transcription quantitative PCR. To this end, the expression levels of *Ltb4r1* mRNA in all bone marrow leukocytes and in bone marrow neutrophils of *Ltb4r1*^ΔPMN^ mice and their wild-type littermates were determined. As expected, *Ltb4r1* mRNA was enriched strongly in the neutrophil population of wild-type mice in comparison with the expression in all leukocyte populations (Figure 5A). The levels of *Ltb4r1* mRNA were reduced pronouncedly in neutrophils of *Ltb4r1*^ΔPMN^ mice with the reduction reaching 95% on average (Figure 5A). We also performed chemotaxis assays to generate additional proof for the functional lack of BLT1 on *Ltb4r1*^ΔPMN^ neutrophils. In line with the reduction of *Ltb4r1* mRNA, chemotaxis toward LTB_4_ and induction of chemokinesis by LTB_4_ were almost nullified in *Ltb4r1*^ΔPMN^ neutrophils (Figure 5B), thus, confirming the gene deletion.

We compared the course of MMP in *Ltb4r1*^ΔPMN^ mice with their wild-type littermates. The development of both cutaneous and mucosal lesions was diminished in *Ltb4r1*^ΔPMN^ mice to the same level as in *Ltb4r1^–/–^* and *Alox5^–/–^* mice (Figure 5). More precisely, the eruption of skin lesions was almost completely abolished in *Ltb4r1*^ΔPMN^ mice (Figure 5, C and D). In line with this clinical phenotype, there was neither recruitment of immune cells into the dermis nor formation of dermal-epidermal clefts, though IgG and C3 were deposited at the dermal-epidermal junction (Figure 5C). Like in the global *Ltb4r1^–/–^* mice, only approximately 50% of *Ltb4r1*^ΔPMN^ mice developed oropharyngeal or conjunctival lesions (Figure 5, D and G). Furthermore, in those *Ltb4r1*^ΔPMN^ mice still developing mucosal lesions, the severity of both oropharyngeal and conjunctival lesions was also reduced (Figure 5, E, F, H, and I).

## Discussion

The pathogenesis of MMP, including the contribution of different immune cell populations infiltrating the dermis, has remained largely elusive. In this study, we further unraveled the pathogenesis of MMP at the molecular and cellular levels by uncovering an essential role of LTB_4_/BLT1 on neutrophils. We first concluded the essential role of LTB_4_/BLT1 in the anti-LAMA332 IgG antibody transfer model from a complete protection of mice globally deficient in *Alox5* and *Ltb4r1* from skin lesions and a marked attenuation in the number of mucosal lesions. Subsequently, we demonstrated that BLT1 is specifically required on neutrophils, thus, providing evidence that the effector functions of neutrophils are crucial for the eruption of mucocutaneous lesions in MMP. Notably, pharmacological inhibition of BLT1 attenuated tissue inflammation even if its administration was initiated after inflammatory lesions were already established, pointing at a continuous requirement of LTB_4_/BLT1 for the progression of disease.

While the protection from inflammatory skin lesions was practically complete in *Alox5* and *Ltb4r1* globally deficient mice as well as in neutrophil-specific *Ltb4r1*-deficient mice, we still detected mucosal lesions in approximately 50% of the genetically deficient mice, albeit with a reduced number of mucosal surface areas affected. At this point, the reasons for this relative difference in the degree of dependence on LTB_4_/BLT1 between the skin and the mucosal surfaces remains elusive. Differences between the skin and mucosal surfaces, e.g., in the strength of adherence between the epidermal and dermal layer, in resident immune sentinel cells initiating inflammatory responses, or in the exposure to mechanical stress promoting the eruption of lesions, might lower the resistance for the eruption of blisters at mucosal surfaces compared with the skin. Also, there may be differences in the expression of laminin 332 in the skin and at mucosal surfaces, possibly leading to differences in the immune response between the skin and the mucosal surfaces. In line with this notion, autoimmunity against laminin 332 more frequently leads to mucosal lesions than to skin lesions (9). Thus, a lower degree of inflammation still emerging in LTB_4_/BLT1 deficiency may be sufficient to elicit mucosal lesions.

Our results give reason to further pursue the development of more effective pharmacological inhibitors of the LTB_4_/BLT1 pathway for use in humans. In that context, it is noteworthy that several promising new compounds for the inhibition of this pathway are currently under development, and the structure of human BLT1 and its physical interactions with LTB_4_ have recently been resolved (25, 26), which will facilitate the development of potent inhibitors of BLT1. Additionally, we have recently demonstrated in the AK801 phase IIa clinical trial that the biopharmaceutical nomacopan, a dual inhibitor of LTB_4_ and the complement factor C5, may be effective in suppressing acute flares of bullous pemphigoid (27). Considering the preclinical results presented here, we suggest that future clinical trials should test the effect of nomacopan in MMP.

Importantly, although meanwhile multiple studies have implicated LTB_4_/BLT1 in the pathogenesis of antibody-induced, neutrophilic tissue inflammation, the mechanisms of how LTB_4_/BLT1 elicit the recruitment of neutrophils into tissues have remained uncertain, among others, because conditional *Ltb4r1* mutants to create cell type–specific *Ltb4r1^–/–^* mice have not been available. BLT1 is, in general, expressed on various leukocyte populations, including neutrophils, eosinophils, monocytes/macrophages, mast cells, dendritic cells, differentiated T cells, and certain subsets of CD4^+^ and CD8^+^ T effector cells (28, 29). The regulation of neutrophil recruitment by LTB_4_/BLT1 may therefore be mediated through a direct effect on neutrophils or through indirect mechanisms involving other immune cell populations, such as tissue-resident immune sentinel cells. To resolve this question in the MMP model, we generated neutrophil-specific *Ltb4r1^–/–^* mice. The resistance of these mice to disease unequivocally revealed that the critical role of LTB_4_ in this model is a direct effect on neutrophils and their migration into the target tissue. Among others, our study also adds to the broader concept of the pathogenesis of diseases featuring autoantibody-induced, neutrophilic tissue inflammation. This group includes models of bullous pemphigoid, bullous pemphigoid–like epidermolysis bullosa acquisita, rheumatoid arthritis, and immune complex–mediated crescentic glomerulonephritis (18, 19, 29–36). In models of all these diseases, tissue inflammation strongly depends on LTB_4_/BLT1. Furthermore, they have in common that the eruption of inflammation depends strongly on the recruitment of neutrophils (29, 32, 37, 38). Notably, except for glomerulonephritis (29), neutrophil recruitment is continuously required to aggravate and sustain full-blown tissue inflammation throughout the course of disease (19, 33, 37). This continued requirement for neutrophil recruitment also explains the continued dependency of disease on LTB_4_/BLT1, which is reflected by the responsiveness of established tissue inflammation to LTB_4_/BLT1 inhibition in these models (33), including the MMP model, as demonstrated here.

The paramount importance of LTB_4_/BLT1 to elicit neutrophil recruitment indicates a nonredundant role of LTB_4_/BLT1 in regulating neutrophil migration into diverse tissue microenvironment. From our point of view, this putative unique position of LTB_4_ in autoantibody-induced tissue inflammation is the result of 2 mechanisms coming together in autoantibody-induced, neutrophilic tissue inflammation: First, LTB_4_ is the only chemoattractant released immediately and in great abundance from neutrophils upon stimulation with immune complexes or the anaphylatoxin C5a. The latter is generated in tissues upon deposition of autoantibodies. This bestows LTB_4_ with the ability to initiate and amplify neutrophil recruitment quickly, as previously suggested (19). Second, it was demonstrated that interstitial tissue migration of neutrophils toward sites of sterile, thermal-induced skin injury as well as to sites of infections with certain pathogens proceeds in a fish school–like formation, i.e., in large, quickly moving, coordinated groups to the inflammatory focus (39–44). This swarm-like migration depends strongly on LTB_4_ as paracrine factor released from neutrophils and directing and amplifying neutrophil migration (39, 40). A plausible explanation for the pivotal role of LTB_4_/BLT1 throughout the different models of antibody-induced tissue inflammation is that, also for the emergence of antibody-induced tissue inflammation, the marked accumulation of neutrophils requires swarm-like migration coordinated by LTB_4_/BLT1.

Collectively, the results of this study highlight neutrophils and the molecular mechanisms orchestrating their migration and actions as potential therapeutic targets for MMP. Our study demonstrates that specifically, the chemoattractant/chemoattractant receptor pair LTB_4_/BLT1 constitute the most promising target in this context. Together, our findings also corroborate the notion that the therapeutic effects of dapsone in MMP may emerge from multiple inhibitory effects on neutrophil activities, including its previously described inhibition of 5-lipoxygenase and BLT1.

## Methods

### Sex as a biological variable.

Our study examined male and female animals, and similar findings are reported for both sexes.

### Mice.

C57BL/6J wild-type, B6.129S2-*Alox5^tm1Fun^/J* (*Alox5^–/–^*), B6.129S4-*Ltb4r1^tm1Adl^*/J (*Ltb4r1^–/–^*), and *MRP8-Cre-ires/GFP* (MRP8-Cre) mice were purchased from The Jackson Laboratory and bred at the University of Lübeck. A conditional knockout of *Ltb4r1* was generated using *Ltb4r1^tm1a(EUCOMM)Hmgu^* embryonic stem (ES) cells (clone H11) purchased from the European Conditional Mouse Mutagenesis Program (EUCOMM). Using the fully verified and karyotyped C57BL/6N ES cells, chimeric founder mice were generated by microinjection into BALB/cJRj blastocysts from Janvier Labs. The resulting mice with the “knockout first allele” were crossed with a C57BL/6N-*Tg(CAG-Flpe)* deleter mouse to remove the selection cassette and create the conditional *Ltb4r1^tm1c^* allele, hereafter referred to as the *Ltb4r1^fl^* allele.

For experiments with the inhibitor CP-105,696, C57BL/6JRj wild-type mice were purchased from Janvier Labs.

PMN-specific *Ltb4r1^–/–^* (*Ltb4r1*^ΔPMN^) mice were generated by crossing homozygous *Ltb4r1^fl/fl^* with MRP8-Cre mice inducing an MRP8 promotor–driven Cre recombination excision event during neutrophil differentiation of precursors. To confirm the gene knockout in neutrophils, bone marrow neutrophils were isolated at the end of the experiments using the Neutrophil Isolation Kit (Miltenyi Biotec), and their RNA was isolated using the RNA Mini Kit (Analytik Jena AG). cDNA was generated using Revert Aid First Strand cDNA Synthesis Kit (Thermo Fisher Scientific). cDNA was used for quantitative PCR using the SYBR Select Mastermix (Thermo Fisher Scientific). Primer pairs used are listed in Supplemental Table 1. Data were acquired using the RealPlex (Eppendorf) cycler.

All experiments were performed in age- and sex-matched mice aged 8–16 weeks. In experiments with *Alox5^–/–^* and *Ltb4r1^–/–^* mice, nonlittermate wild-types and in experiments with *Ltb4r1*^ΔPMN^ mice wild-type littermates served as controls.

All mice used in this project were kept under specific pathogen–free conditions according to Federation of European Laboratory Animal Science Associations recommendations and were bred and maintained in a barrier rodent facility of the University of Lübeck.

Groups of 2–5 females and males were housed in Greenline GM 500 cages (Tecniplast, Thermo Fisher Scientific) lined with 4HK-13Kg Aspen bedding (Tapvei) and enriched with nesting material (Ssniff) in a photoperiod of 12 hours of light and 12 hours of dark. Except for the experiments comparing global gene knockouts, the different experimental groups were housed together in the same cages to minimize cage effects. Food pellets (food type 1314) from Altromin and tap water were available ad libitum.

### Conduct of the antibody transfer model of anti-LAMA332 MMP and treatment with CP-105,696.

The antibody transfer model of anti-LAMA332 MMP was induced, as previously described (18, 45). Briefly, total IgG of anti-mouse laminin 332 IgG was isolated by Protein G affinity chromatography from sera of New Zealand white rabbits that had been immunized with protein fragments of the α3 chain (Eurogentec). Mice were injected subcutaneously into the neck with 6 mg of anti-LAMA332 every other day until day 10. The percentage of the total body surface affected by such lesions was determined every 3 days starting on day 4. At the end of the experiment on day 16, the oral cavity, the pharynx, the larynx, and the esophagus were inspected for blisters and erosions by endoscopy (Hopkins Optik 64019BA).

The BLT1 antagonist CP-105,696 (Sigma-Aldrich), dissolved in 10% ethanol, 0.5% methylcellulose, and 0.5% Tween 80, was administered by oral gavage daily at a dose of 1, 5, or 10 mg/kg, as previously described (36).

The mucosal involvement in the oral cavity was benchmarked using a scoring system from 0 to 4, as previously described (16). Briefly, the oral cavity was separated into 4 quarters, and for each quarter affected by inflammatory lesions, 1 score point was given.

### (Immuno-)histopathology.

Biopsies of perilesional skin, i.e., skin directly adjacent to active inflammatory skin lesions, and of palpebral conjunctiva were fixed in 4% Histofix solution (Carl Roth GmbH). Biopsies were embedded in paraffin and cut to 6 μm sections. For histopathology, sections were stained with H&E. Ly-6G staining was performed on deparaffinized sections of perilesional skin. Antigens were retrieved using the Digest-All 3 pepsin solution (Thermo Fisher Scientific) for 10 minutes at room temperature (RT). Slides were washed with 0.01 M Tris-buffered saline (pH 7.6) and blocked with 5% normal goat serum (NGS) for 1 hour prior to the application of rat anti-mouse Ly-6G antibody (BioLegend; 1:100; 127603) in 5% (vol/vol) NGS application at 4°C overnight. After washing, sections were incubated with Alexa Fluor 488 AffiniPure Goat anti-rabbit IgG (Jackson ImmunoResearch; 1:500; 111-545-144) for 1 hour at RT, washed again, and mounted with DAPI Fluoromount-G (SouthernBiotech). Immunofluorescence staining for IgG and complement C3 was performed as previously described (46). Briefly, 6 μm cryosections of perilesional skin were washed with 0.01 M PBS (pH 7.2), fixed with acetone, and stained with Alexa Fluor 594 AffiniPure Goat Anti-Rabbit IgG (Jackson ImmunoResearch; 1:500; 112-585-167) or purified rat anti-mouse complement C3 (Cedarlane Labs; 1:200; CL7503AP) for 1 hour at RT. Sections to be stained for C3 were washed and incubated with Alexa Fluor 488 AffiniPure Goat anti-rabbit IgG for 1 hour at RT. Slides were washed and mounted with DAPI Fluoromount-G. All images were acquired with the Keyence HS All-in One Fluorescence Microscope (Keyence GmbH) at 200× original magnification.

### Assessment of conjunctivitis.

To assess conjunctivitis, paraffin biopsies of the palpebral conjunctiva were processed and stained with H&E, as described above. Each sample was cut at 3 levels, visualized using the Keyence HS All-in One Fluorescence Microscope at 200× original magnification, and evaluated for subepidermal split formation using the BZ II analyzer (Keyence GmbH). The severity of palpebral conjunctivitis was assessed by measuring the length of the largest split and applying the following scoring system: 0, no split; 1, <100 μm; 2, 100–199 μm; 3, 200–299 μm; 4, >299 μm.

### Immune cell infiltration analysis through flow cytometry.

To describe the immune cell infiltrate in the skin, 6 mm biopsies from lesional skin, i.e., inflamed skin, and perilesional skin, i.e., skin directly adjacent to inflamed skin sites, were collected at the end of the experiment and processed into cell suspensions. Cell viability was determined using Viobility 405/452 Fixable Dyes (Miltenyi Biotec). Cells were blocked using mouse FcR Blocking Reagent (Miltenyi Biotec). Staining proceeded with a master mix of antibodies against CD45 (rat anti-CD45 FITC; 1:100; BioLegend; 553080), CD11b (rat anti-CD11b Brilliant Violet 650; 1:100; BioLegend; 101259), Ly-6G (rat anti-Ly-6G PerCP Vio 700; 1:10; Miltenyi Biotec; 130-103-793; and rat anti-Ly-6G APC/Cy7; 1:100; BioLegend; 127624), F4/80 (rat anti-F4/80 PE; 1:100; BioLegend; 123110), and Siglec-F (rat anti–Siglec-F APC/Cy7; 1:100; BioLegend; 565527). The manufacturers and fluorochromes of these antibodies are compiled in Supplemental Table 2. Cells were fixed in 4% Histofix. To determine the number of cells, Count Bright Absolute Counting Beads (Invitrogen) were added to each sample, and cell numbers were calculated according to the manufacturer’s instruction. Data were acquired on the CytoFLEX S Flow Cytometer (Beckman Coulter) and analyzed with the corresponding CytExpert2.4 Software (Beckman Coulter). The cell populations were gated as shown in Supplemental Figure 1.

### Chemotaxis assays.

Bone marrow neutrophils, freshly isolated, as described above, were resuspended to a 10^6^ cells/mL cell suspension. A total of 10^5^ cells were seeded onto 3 μM pore–sized Transwell inserts of 24-well plates (Sarstedt). A total of 3.3 ng/mL LTB_4_ was added to the bottom wells (chemoattractant gradient) or into the upper well (chemokinesis control). No LTB_4_ was added to the migration controls. The loaded Transwell plates were incubated for 2.5 hours at 37°C, 5% CO_2_. Afterward, cells were collected from the bottom wells and counted on the CytoFLEX S Flow Cytometer, and the percentage of the cells that migrated into the bottom well was calculated.

### Statistics.

All data are presented as mean ± SEM. Disease severity scores for skin inflammation were compared by 2-way ANOVA with Holm-Šídák multiple comparisons test. Endoscopic and conjunctiva scores were analyzed by Mann-Whitney *U* test or Kruskal-Wallis test with Dunn’s correction for multiple comparisons depending on if 2 or more groups were compared. Contingency tables were compared by χ^2^ test. Cell numbers in the skin and *Ltb4r1* mRNA expression levels were compared by 2-way ANOVA with Holm-Šídák multiple comparisons test. *P* < 0.05 was considered statistically significant throughout the study. All calculations were performed using GraphPad Prism 10.

### Study approval.

All experimental procedures in animals were evaluated and approved by the animal protection authority of the Schleswig-Holstein state government, Kiel, Schleswig-Holstein, Germany.

### Data availability.

Values for all data points found in graphs are in the Supporting Data Values file.

## Author contributions

CDS planned the study and provided funding for the study. TB, SM, SP, PS, MN, SG, JP, and WP conducted experiments for the paper. TR generated the *Ltb4r1*-floxed mouse line used in this study. TB, SM, ES, TR, and CDS analyzed the results. CDS, TB, SM, SP, and TR all wrote parts of the manuscript. All authors edited the manuscript. TB and SM contributed equally to this publication in the quantity of data generated and analyzed. TB was involved in the initial planning and conduct of the project and was therefore assigned the first position of the author list.

## Supplementary Material

Supplemental data

Supporting data values

## Version 1 09/23/2025

Electronic publication
