# Supplementary material for: The receptor BLT1 is essential on neutrophils in a mouse model of mucous membrane pemphigoid
Source: JCI Insight. 2025 Sep 23;10(18):e173914. doi: 10.1172/jci.insight.173914 (PMC12487858; doi:10.1172/jci.insight.173914)
Supplement: Supplemental data [file jciinsight-10-173914-s033.pdf]

Supplementary figure S1 - Gating Strategy

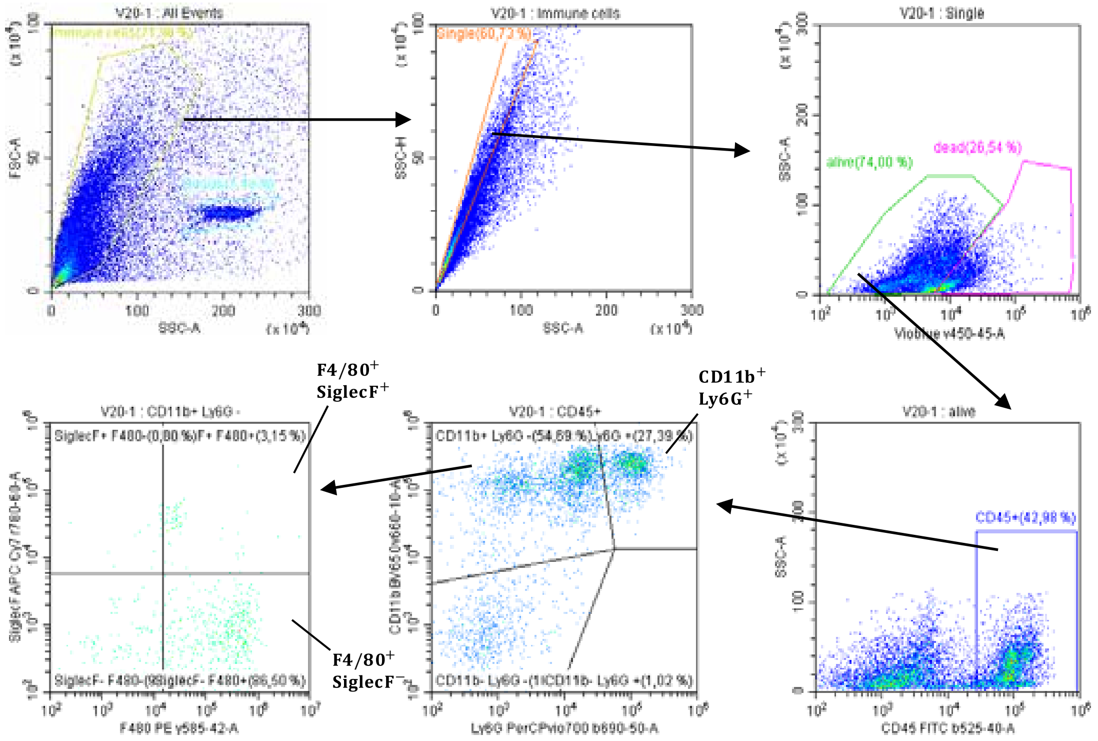

Supplementary figure S1. Gating strategy applied to distinguish select cell populations in skin lysates.

Supplementary Table S1.

Primer name	Target gene	Primer Sequence 5’-3’
m.Blt1 - forward	*Ltb4r1*	AGGACCCTGGCACTAAGACA
m.Blt1 - reverse		AGCCATCAAAAGGACAGGGT
m.HPRT-forward	*Hprt1*	GGTTAAGCAGTACAGCCCCA
m.HPRT- reverse		CAAATCCAACAAAGTCTGGCCT

Supplementary table S2. Antibodies used to characterized dermal infiltrates by flow cytometry.

Fluorochrome	Specificity	Company
FITC	CD45	BD Biosciences
Brilliant Violet 650	CD11b	BioLegend
PerCP Vio700	Ly-6G	Miltenyi Biotec
PE	F4/80	BioLegend
APC/Cy7	Siglec-F	BioLegend
